# Risks of Pneumonia in COPD Patients with New-Onset Atrial Fibrillation

**DOI:** 10.3390/jcm7090229

**Published:** 2018-08-21

**Authors:** Ya-Hui Wang, Chih-Cheng Lai, Cheng-Yi Wang, Hao-Chien Wang, Chong-Jen Yu, Likwang Chen, On Behalf of the Taiwan Clinical Trial Consortium for Respiratory Diseases

**Affiliations:** 1 Medical Research Center, Cardinal Tien Hospital and School of Medicine, College of Medicine, Fu-Jen Catholic University, New Taipei City 23141, Taiwan; yhwang531@gmail.com; 2 Department of Intensive Care Medicine, Chi Mei Medical Center, Liouying 73657, Taiwan; dtmed141@gmail.com; 3 Department of Internal Medicine, Cardinal Tien Hospital and School of Medicine, College of Medicine, Fu-Jen Catholic University, No.362, Zhongzheng Road, Xindian District, New Taipei City 23148, Taiwan; 4 Department of Internal Medicine, National Taiwan University Hospital and College of Medicine, National Taiwan University, Taipei 10048, Taiwan; jefferycjyu@ntu.edu.tw; 5 Institute of Population Health Sciences, National Health Research Institutes, Zhunan 35053, Taiwan; likwang.chen@gmail.com

**Keywords:** atrial fibrillation, chronic obstructive pulmonary disease, pneumonia, risk

## Abstract

The association between Atrial Fibrillation (AF) and pneumonia remains unclear. This study aims to assess the impact of AF on high pneumonia risk group—chronic obstructive pulmonary disease (COPD)—In order to find the association between AF and the risk of pneumonia. The COPD cohort was extracted from National Health Research Institute of Taiwan. The AF cohort comprised all COPD patients with new-onset AF (International Classification of Diseases (ICD)-9 code 427.31) after COPD diagnosis. We further sampled non-AF cohort and performed 1:1 propensity score matched analysis to improve the balance of baseline characteristics between AF and non-AF cohort. The outcomes were pneumonia and pneumonia requiring mechanical ventilation (MV). From 2000–2011, a total of 6228 patients with COPD and AF, and matched 84,106 control subjects were enrolled. After propensity score matching, we identified 6219 patients, each with AF, and matched controls without AF. After propensity score matching, the AF cohorts had higher risk of mortality (adjusted hazard ratio (aHR), 1.24; 95% confidence interval (CI), 1.15–1.34), pneumonia (aHR, 1.17; 95% CI, 1.07–1.27), and pneumonia requiring MV (aHR, 1.33; 95% CI, 1.18–1.50) in comparison with the matched non-AF cohort. After adjusting for mortality from causes other than outcomes of interest as a competing risk, AF remains significantly associated with pneumonia and pneumonia requiring MV. The risks of pneumonia were higher in this population with AF than in those without AF, and the risk was still significant after the adjustment for the competing risk of all-cause mortality.

## 1. Introduction

Pneumonia is one of the most common types of infections, and it is associated with high morbidity and mortality. The risk factors of pneumonia include smoking, recent viral respiratory tract infection, elderly patients, difficulty swallowing due to neurologic disease, immunocompromised status, recent trauma or trauma, heart diseases, and chronic lung diseases, such as chronic obstructive pulmonary disease (COPD) [1,2,3]. To face this clinical entity, it is important to identify the population at high risk, such as vaccination for elderly and COPD patients.

Atrial Fibrillation (AF) is the most common type of cardiac arrhythmia and both its prevalence and incidence are increasing worldwide [4,5,6]. Furthermore, AF can cause significant morbidity and mortality, and AF-associated morality increased by 1.9 to 2-fold from to 1990–2010 [4]. All these findings suggest that AF has become a global burden on public health. AF is traditionally reported to be associated with cardiovascular diseases; however, an increasing number of studies [7,8] have shown the significant association between AF and non-cardiovascular diseases such as cancer [9,10], sepsis [11,12,13], obstructive sleep apnea [14,15], chronic kidney disease [16,17,18], and chronic obstructive pulmonary disease (COPD) [19,20,21,22,23,24].

Moreover, one recent study found that AF itself is an independent risk factor for hospital-acquired pneumonia [25]. However, the impact of AF on the development of subsequent pneumonia among patients at high risk is unclear. Patients with COPD are prone to respiratory infections; therefore, we hypotheses that AF may be associated with increasing risk of pneumonia in this vulnerable population. To clarify this issue, we conduct this study to assess the impact of new-onset AF on the patients with COPD, and to find out the association between AF and incidence of pneumonia. 

## 2. Methods

### 2.1. Data Source

We used the database constructed by the NHRI (National Health Research Institute) of Taiwan. This database includes outpatient visits, hospital admissions, prescriptions, and disease and vital status data for 99% of the population (23 million people) in Taiwan. The NHRI used original reimbursement data from the National Health Institute (NHI) database to construct a longitudinal database of COPD patients from 1998 to 2010. This cohort included 2,200,000 patients representing 60.5% of all patients with heart or lung disease in the NHI database (*n* = 3,635,539). The patient records and information were anonymized and de-identified prior to analysis. Therefore, informed consent was not required and was specifically waived by the Institutional Review Board. Ethics approval was obtained from the Institutional Review Board of Cardinal Tien Hospital (IRB No.: CTH-106-3-5-058). 

### 2.2. Study Cohort

The COPD cohort was extracted from National health insurance research database (NHIRD). All patients aged between 40 and 100 years, who had experienced a hospital admission or at least three outpatient visits with a COPD diagnostic code within one year from 1 January 2000 to 31 December 2010 were identified. COPD diagnoses were confirmed by the International Classification of Diseases, Ninth Revision (ICD-9) codes 491, 492, or 496. Patients were excluded for the following reasons: (1) incomplete demographic data, (2) had not undergone a lung function test within one year before or after the COPD diagnosis, and (3) had not received a COPD diagnosis after the lung function test. We also excluded those who were dead or diagnosed with AF prior to being indexed. Overall, 90,334 COPD patients were included in this study cohort.

**Figure 1:**
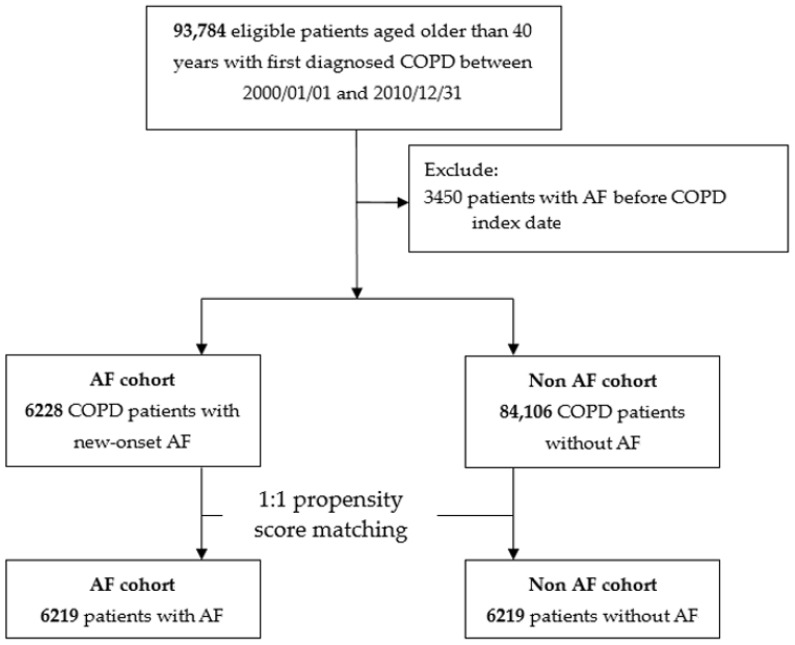
Study flow chart: a population-based cohort study.

The AF cohort comprised all COPD patients with new-onset AF (ICD-9 code 427.31) after COPD diagnosis. The index date was defined as the date of new-onset AF diagnosis. Index dates for subjects in the control group were randomly assigned dates of medical records. We further sampled non-AF cohort and performed 1:1 propensity score matched analysis to improve the balance of baseline characteristics between AF and non-AF cohort. In the non-AF cohort, patients who had previous history of AF were also excluded. A propensity score analysis was used to reduce potential confounding caused by unbalanced covariates. The propensity score, i.e., the probability of having AF was estimated using a logistic regression model conditional on the covariates of the time from COPD diagnosis to index date, age, gender, index year of AF, monthly income, hospital level, severe exacerbations of COPD in one year prior to index date (never, 1, or ≥2 times/year), medications for COPD, medications for hypertension, other medications and individual comorbidities. Finally, there are 6219 cases with AF and 6219 matched controls in this study (Figure 1).

### 2.3. Demographic Characteristics and Comorbidities

Baseline demographic characteristics, including age, gender, monthly income (less than NT$ 19,100, NT$ 19,100–NT$ 41,999, and more than NT$ 42,000), hospital level (medical center, regional, district, and others), years from COPD diagnosis to index date, severe exacerbations of COPD in one year prior to index date (never, 1, or ≥2 times/year), and the index year of AF (2000–2011) were extracted. Comorbidity data were retrieved according ICD-9, and these involved sleep apnea, myocardial infarction, congestive heart failure, peripheral vascular disease, cerebrovascular disease, dementia, rheumatologic disease, peptic ulcer disease, hemiplegia or paraplegia, renal disease, moderate/severe liver disease, tumor and diabetes. For each patient, the Charlson Comorbidity Index (CCI) was used to determine severity of comorbidities. Additionally, the CHA2DS2-VASc (Congestive Heart Failure, Hypertension, Age ≥75 [Doubled], Diabetes Mellitus, Prior Stroke or Transient Ischemic Attack [Doubled], Vascular Disease, Age 65–74, Female) score and CHADS2 score were calculated for each of the subjects [26]. CHA2DS2-VASc/CHADS2/CCI scores are commonly used to evaluate the severity of AF and AF-associated diseases, such as stroke. Concomitant medications such as aspirin, clopidogrel, ticlopidine, dipyridamole, nitrate, statin, nonsteroidal anti-inflammatory drugs, anti-hyperglycemic drugs, proton-pump inhibitor (PPI), medication for hypertension (alpha-blocker, beta-blocker, calcium-channel blocker, diuretic, and angiotensin-converting-enzyme inhibitor/angiotensin II receptor blockers) and medication for COPD (long-acting beta agonist (LABA), Short-acting β2-agonist (SABA), long-acting muscarinic antagonist (LAMA), inhaled corticosteroid (ICS)), were recorded. 

### 2.4. Outcomes

The outcomes were pneumonia (ICD-9-CM codes 480–486, and 507), pneumonia requiring invasive and non-invasive mechanical ventilation (MV) (as presentation for severe pneumonia), and all-cause mortality. Because of the high mortality rate and older-aged in COPD patients, competing risk analysis using the Fine and Gray model was also performed [24]. All subjects were followed until the occurrence of events of interest, death or the end of the study (31 December 2011).

### 2.5. Statistical Analysis

Descriptive statistics were used to characterize the study population at baseline. Continuous variables were presented as mean ± SD; categorical variables were described as counts and percentages. Baseline characteristics were compared between groups using Chi-square tests for categorical variables and independent *t*-tests for continuous variables. *p* value < 0.05 was considered to indicate statistical significance. Cox regression models were used to calculate the crude and adjusted hazard ratios (HRs) of different outcomes in the two study cohorts. Adjusted HRs and 95% confidence intervals (CIs) were calculated using Cox regression models adjusted for propensity scores (continuous), cardioversion procedure, and amiodarone use. Amiodarone use was calculated as a time-varying covariate. The non-AF cohort was selected as the reference group. The crude incidence rate of different outcomes was calculated as the total number of events during the follow-up period, divided by person-years at risk. The competing risk analysis and subgroup analysis were performed to further assess the robustness of our study findings. We applied the Fine and Gray competing risk model [27] to derive sub-distribution hazard ratios and 95% CIs in relation to the primary outcomes. We used SAS software version 9.4 (SAS Institute Inc., Cary, NC, USA) for data analysis. 

## 3. Results 

### 3.1. Characteristics of the Study Population

During the study period, a total of 6228 patients with COPD and AF, and matched 84,106 control subjects were enrolled (Figure 1). Table 1 summarized the demographic characteristics of these groups. AF cohorts were older, longer duration between COPD diagnosis year and index date of AF and more male than control group (all *p* < 0.05). In addition, significant differences regarding distribution of monthly income, hospital level, the number of COPD with severe acute exacerbation (AE), anti-hypertension medication, most of the commonly used cardiovascular medication and almost all of COPD inhaled and oral drugs except LABA, were noted between AF cohort and control group (all *p* < 0.05). Additionally, AF cohorts had higher CCI, more myocardial infarction, congestive heart failure, peripheral vascular disease, cerebrovascular diseases, dementia, peptic ulcer disease, renal disease, liver diseases, cancer, and diabetes mellitus than the control group (all *p* < 0.05). The AF group had higher CHA2DS2-VASc score and CHADS2 score than the control group (both *p* < 0.05). After propensity score matching, we identified each of the 6219 patients with AF, and matched controls with similar characteristics including age, gender, duration from COPD diagnosis to index date, income, hospital level, all of the medications for COPD, hypertension, cardiovascular diseases and baseline comorbidities. 

### 3.2. Risk of Death and Pneumonia

During the follow-up period, the AF cohorts had higher risk of pneumonia (aHR, 1.17; 95% CI, 1.07–1.27), pneumonia requiring MV (aHR, 1.33; 95% CI, 1.18–1.50), and all-cause mortality (aHR, 1.24; 95% CI, 1.15–1.34), in comparison with the non-AF cohorts (Table 2). After adjusting for all-cause mortality from causes other than outcomes of interest as a competing risk, AF was significantly associated with pneumonia (HR, 1.12; 95% CI, 1.03–1.22) and pneumonia requiring MV (HR, 1.15; 95% CI, 1.03–1.28).

### 3.3. Association between CHA2DS2-VASc/CHADS2/CCI Scores and Risk of Pneumonia in COPD Patients with AF

The overall incidence of pneumonia was 19.22 per 100 person-years (3014 events per 15,685 person-year) during the follow-up period (Table 3). The incidences of pneumonia were 9.31 and 51.31 per 100 person-years for patients with a CHA2DS2-VASc score of 0 and 9, respectively. CHA2DS2-VASc score ≥2 significantly increases the risk of pneumonia relative to score of 0 (aHR, 1.54; 95% CI, 1.09–2.18 for score 2; aHR, 1.84; 95% CI, 1.31–2.59 for score 3; aHR, 1.93; 95% CI, 1.37–2.72 for score 4; aHR, 2.13; 95% CI, 1.51–3.00 for score 5; aHR, 2.82; 95% CI, 1.99–3.99 for score 6; aHR, 2.65; 95% CI, 1.85–3.81 for score 7; aHR, 3.63; 95% CI, 2.39–5.51 for score 8; aHR, 5.41; 95% CI, 2.68–10.91 for score 9). This trend was also noted when death was treated as a competing risk in the Fine and Gray competing risk model. Additionally, the incidences of pneumonia were 12.21 and 46.49 per 100 person-years for patients with a CHA2DS2 score of 0, and 6, respectively. CHA2DS2 score ≥2 significantly increases the risk of pneumonia relative to score of 0 (aHR, 1.43; 95% CI, 1.15–1.77 for score 2; aHR, 1.64; 95% CI, 1.32–2.04 for score 3; aHR, 1.79; 95% CI, 1.43–2.24 for score 4; aHR, 2.11; 95% CI, 1.68–2.64 for score 5; aHR, 3.18; 95% CI, 2.37–4.25 for score 6) and trend was also noted when death was treated as a competing risk in the Fine and Gray competing risk model. We found that CCI score was correlated with the risk of pneumonia (aHR, 1.29; 95% CI, 1.18–1.42 for score 2–3; aHR, 1.80; 95% CI, 1.56–2.08 for score ≥4 compared to a score of 1), even when death was treated as a competing risk. 

### 3.4. Association between COPD Medications and Risk of Pneumonia in COPD Patients

Table 4 shows the subgroup analysis of risk of pneumonia among COPD patients with AF, compared to matched non-AF cohort. After adjusted for propensity score, cardioversion procedure, and time-dependent of amiodarone use, tests of interactions were not significant for use of LABA (*p* = 0.558), use of SABA (*p* = 0.358), use of LAMA (*p* = 0.920) and use of ICS (*p* = 0.262). 

## 4. Discussion

This national population-based study, compromising two matched cohorts each comprising 6219 COPD patients with or without new onset of AF has several significant findings. There were no other differences in these two cohorts except AF or not. We found that the AF cohorts had a higher risk of mortality, pneumonia, and pneumonia requiring MV, in comparison with the matched cohort without AF. There was a consistent and negative effect of AF in pneumonia, even after adjusting for mortality from causes other than outcomes of interest as a competing risk. The negative impacts of AF were shown in subgroup analysis. None of the subgroups investigated appeared to modify the effect of AF on patients’ outcomes.

The positive impact of AF on the outcomes in this study is consistent with previous studies [19,21]. In the analysis of Northern California Kaiser Permanente Medical Care Program [19], patients with AF had a higher risk of hospitalization than patients without AF (relative risk: 1.98, 95% CI: 1.73–2.25). Another study [21] had shown that AF could be an independent risk factor of death (OR: 2.66, 95%: 1.39–5.09). In this study, we have used a nationwide population-based cross-sectional study that covers 99.0% of Taiwan’s population and contains nearly complete follow-up information for the whole study population. In this study, we used propensity score matching to minimize the effects of possible confounding variables. Therefore, our findings should be representative and could be generalized. In summary, all of these should indicate that AF can directly affect the prognosis, and these results have clinical implications. In the clinical condition of increasing burden of high-risk groups in the whole word and AF remaining the common arrhythmia among them, we should devote more effort to identifying the patients with AF. After early diagnosis of AF in high-risk patients, we may give appropriate treatment and control for AF, and further ameliorate the negative effect of AF.

Inflammation may be the mechanism with accumulating evidence that progression of comorbidities is associated with AF. Theoretically, inflammation plays an important role in AF patients [28,29,30]. Currently, an increasing amount of evidence suggests that inflammation may participate in the onset and continuation of AF and AF-associated thrombosis through endothelial dysfunction, production of tissue factor, increase in the activation of platelet and increase fibrinogen expression [30,31]. Various inflammatory markers such as C-reactive protein (CRP), tumor necrosis factor (TNF)-α, interleukin (IL)-2, IL-6, IL-8, and monocyte chemoattractant protein (MCP)-1 have been demonstrated to be associated with AF [31,32]. Besides this, for high-risk patients (such as COPD in this study), persistent and systemic inflammation is considered to play a significant role in its pathogenesis [29,30,31,32,33]. Elevated levels of CRP, IL-6, IL-8, and TNF-α have been reported in patients with COPD [34,35,36]. Currently, there is some discussion about the association between inflammation and the respiratory tract microbiome [37,38]. Some byproducts of inflammation may serve as growth factors for bacteria. This may contribute to the incidence of pneumonia [38]. 

Besides, several other factors may help to explain the effect of AF on COPD patients. Clinically, agents used to treat COPD, including beta-adrenergic agonists and theophylline can result in tachyarrhythmia [39]. In contrast, medication used for controlling AF, such as sotalol, propafenone and non-selective-β-blockers may cause bronchospasm [39]. In addition, the symptoms of COPD patients could be worse due to AF associated with irregular heart beat and reduced diastolic filling of the ventricle [20]. Thus, it is difficult to control COPD when the patients have COPD and AF at the same time. Therefore, it is possible that AF and COPD share the common pathway of inflammation, and interact between themselves through a similar mechanism.

CHA2DS2-VASc [40], CHADS2 [41] and CCI scores [42] were applied in this study. In all three scoring systems, patients with higher scores are at higher risk of pneumonia and pneumonia with MV. CHA2DS2-VASc and CHADS2 are used to calculate the risk of stroke in AF patients. CCI score is a score system to evaluate disease severity. In this study, all three systems work well to find high-risk groups. However, CHA2DS-VASc has better ability than the other two scoring systems. Maybe CHA2DS2-VASc score (Congestive heart failure (1 point), Hypertension (1 point), Age ≥75 (2 points), Diabetes (1 point), Stroke or TIA (2 points), Vascular disease (1 point), Age 65–74 (1 point), Female sex (1 point)) cover more risk factors and age ranges. 

Nevertheless, several inherent limitations must be considered. First, as with all claims databases, the data describing lifestyle factors such as body mass index and smoking are not available. Second, we defined AF by ICD-9 codes from administrative data reported by physicians. Although the diagnostic accuracy of AF has been validated in previous studies [43,44], this issue remains a concern. Third, the data of pulmonary function test and the clinical symptoms and signs were not available from NHIRD database. However, here we offer two matched groups without differences except AF or not. We also have tried to adjust the commonly used medications for COPD including LABA, LAMA, SABA, and ICS, and the frequency of AE between COPD patients with and without AF. Therefore, we almost match the severity of COPD in these two groups. 

## 5. Conclusions

The risk of pneumonia was higher in patients with AF than in those without AF, and the risk was still significant after the adjustment for the competing risk of all-cause death. Thus, the net clinical benefit of pneumonia prevention for high-risk patients with AF needs to be emphasized, especially considering the impact of the high mortality burden. It is noteworthy that our results stressed the need for paying attention to new-onset AF in high-risk patients.

## Figures and Tables

**Table 1:** Demographic and clinical characteristics of the chronic obstructive pulmonary disease (COPD) patients with and without Atrial Fibrillation (AF).

Variables	Non-AF Cohort		AF Cohort	
Patient (no.)	6219		6219	
Years from COPD diagnosis to index date	3.61 ± 3.05		3.65 ± 3.10	
Age (year)	71.16 ± 9.66		71.15 ± 9.47	
Male gender	4560	73.32%	4518	72.65%
Index year of AF				
2000	223	3.59%	196	3.15%
2001	374	6.01%	379	6.09%
2002	410	6.59%	415	6.67%
2003	418	6.72%	410	6.59%
2004	550	8.84%	555	8.92%
2005	534	8.59%	598	9.62%
2006	569	9.15%	500	8.04%
2007	582	9.36%	610	9.81%
2008	670	10.77%	627	10.08%
2009	544	8.75%	591	9.50%
2010	733	11.79%	696	11.19%
2011	612	9.84%	642	10.32%
Monthly income, *n* (%)				
<19,100	2489	40.02%	2462	39.59%
19,100–41,999	3047	49.00%	3063	49.25%
≥42,000	683	10.98%	694	11.16%
Hospital level, *n* (%)				
Level 1	2171	34.91%	2113	33.98%
Level 2	2339	37.61%	2416	38.85%
Level 3	1383	22.24%	1375	22.11%
Level 4 (rural area)	326	5.24%	315	5.07%
COPD severe AE				
0	3036	48.82%	2996	48.17%
1	1060	17.04%	1090	17.53%
≥2	2123	34.14%	2133	34.30%
Medication for COPD				
LABA	132	2.12%	129	2.07%
SABA	1046	16.82%	1017	16.35%
LAMA	395	6.35%	387	6.22%
ICS	1228	19.75%	1220	19.62%
Medication for hypertension				
Alpha-Blocker	877	14.10%	908	14.60%
Beta-Blocker	2663	42.82%	2645	42.53%
Calcium-Channel Blocker	3889	62.53%	3839	61.73%
Diuretic	3629	58.35%	3606	57.98%
ACEi or ARB	3130	50.33%	3142	50.52%
Other medication				
Aspirin	1252	20.13%	1332	21.42%
Clopidogrel	518	8.33%	532	8.55%
Ticlopidine	221	3.55%	248	3.99%
Dipyridamole	1505	24.20%	1498	24.09%
Nitrate	153	2.46%	166	2.67%
Statin	733	11.79%	772	12.41%
NSAID	4903	78.84%	4913	79.00%
Anti-hyperglycemic drugs	1310	21.06%	1305	20.98%
Proton-pump inhibitor	926	14.89%	911	14.65%
Baseline comorbidities				
Charlson score	1.98 ± 1.24		1.99 ± 1.21	
Sleep apnea	14	0.23%	26	0.42%
Old myocardial infarction	194	3.12%	179	2.88%
Congestive heart failure	1251	20.12%	1322	21.26%
Peripheral vascular disease	58	0.93%	63	1.01%
Cerebrovascular disease	553	8.89%	562	9.04%
Dementia	206	3.31%	202	3.25%
Rheumatologic disease	47	0.76%	41	0.66%
Peptic ulcer disease	1100	17.69%	1069	17.19%
Hemiplegia or paraplegia	8	0.13%	8	0.13%
Renal disease	263	4.23%	297	4.78%
Moderate/Severe liver disease	232	3.73%	207	3.33%
Tumor	273	4.39%	284	4.57%
Diabetes	902	14.50%	896	14.41%

COPD = chronic obstructive pulmonary disease; AF = atrial fibrillation; AE = exacerbation; LABA = long-acting beta agonist; SABA = short-acting beta-agonists; LAMA = long-acting muscarinic antagonist; ICS = inhaled corticolsteroid; ACEi = angiotensin-converting-enzyme inhibitor; ARB = angiotensin II receptor blocker; NSAID = nonsteroidal anti-inflammatory drug.

**Table 2:** Incidences and risks of pneumonia, pneumonia with mechanical ventilator (MV), and all-cause mortality among COPD patients with and without AF after propensity score matching.

Outcome	Crude	Adjusted	Competing Risk
	HR (95% CI)	HR ^a^ (95% CI)	HR ^a^ (95% CI)
Mortality	1.28 (1.19, 1.37)	1.24 (1.15, 1.34)	-
Pneumonia	1.65 (1.54, 1.76)	1.17 (1.07, 1.27)	1.12 (1.03, 1.22)
Pneumonia with MV	1.78 (1.62, 1.97)	1.33 (1.18, 1.50)	1.15 (1.03, 1.28)

^a^ Adjusted for propensity score (continuous), cardioversion procedure, and amiodarone use, which was calculated as a time-varying covariate.

**Table 3:** Incidence rates and risks of pneumonia in COPD patients with AF.

Scores	Pneumonia			Crude	Adjusted	Competing Risk
	#Event	PY ^a^	IR ^b^	HR (95% CI)	HR ^c^ (95% CI)	HR ^c^ (95% CI)
CHA2DS2-VASc						
0	52	559	9.31	Reference	Reference	Reference
1	192	1587	12.10	1.26 (0.93, 1.72)	1.28 (0.89, 1.86)	1.25 (0.87, 1.81)
2	395	2815	14.03	1.41 (1.06, 1.88)	1.54 (1.09, 2.18)	1.46 (1.03, 2.07)
3	533	3095	17.22	1.64 (1.23, 2.18)	1.84 (1.31, 2.59)	1.68 (1.20, 2.37)
4	595	2864	20.78	1.86 (1.40, 2.47)	1.93 (1.37, 2.72)	1.68 (1.19, 2.36)
5	515	2356	21.86	1.90 (1.43, 2.53)	2.13 (1.51, 3.00)	1.79 (1.27, 2.52)
6	397	1410	28.15	2.31 (1.73, 3.09)	2.82 (1.99, 3.99)	2.34 (1.65, 3.31)
7	239	786	30.41	2.36 (1.75, 3.19)	2.65 (1.85, 3.81)	2.11 (1.47, 3.04)
8	85	191	44.53	3.00 (2.12, 4.23)	3.63 (2.39, 5.51)	2.69 (1.77, 4.08)
9	11	21	51.31	2.98 (1.55, 5.71)	5.41 (2.68, 10.91)	3.51 (1.74, 7.08)
*p* for trend test	<0.0001					
CHADS2 Score						
0	146	1195	12.21	Reference	Reference	Reference
1	447	3472	12.88	1.05 (0.87, 1.26)	1.12 (0.90, 1.41)	1.10 (0.88, 1.38)
2	714	4096	17.43	1.30 (1.09, 1.56)	1.43 (1.15, 1.77)	1.34 (1.08, 1.66)
3	656	3039	21.58	1.49 (1.25, 1.78)	1.64 (1.32, 2.04)	1.42 (1.14, 1.76)
4	506	2113	23.94	1.61 (1.34, 1.94)	1.79 (1.43, 2.24)	1.58 (1.27, 1.98)
5	427	1516	28.17	1.82 (1.50, 2.19)	2.11 (1.68, 2.64)	1.78 (1.42, 2.24)
6	118	254	46.49	2.40 (1.88, 3.06)	3.18 (2.37, 4.25)	2.44 (1.82, 3.26)
*p* for trend test	<0.0001					
CCI score						
1	1216	7865	15.46	Reference	Reference	Reference
2–3	1437	6712	21.41	1.28 (1.19, 1.38)	1.29 (1.18, 1.42)	1.23 (1.12, 1.34)
≥4	361	1108	32.57	1.67 (1.48, 1.88)	1.80 (1.56, 2.08)	1.47 (1.28, 1.70)
*p* for trend test	<0.0001					

^a^ Person-Years, ^b^ Incidence rate (per 100 person-years), ^c^ Adjusted for propensity score (continuous), cardioversion procedure, and amiodarone use, which was calculated as a time-varying covariate. # indicates number of pneumonia cases.

**Table 4:** Subgroup analysis of risk of pneumonia among COPD patients with AF and matched non-AF cohort.

Subgroups	Crude	*p* Value	Adjusted	*p* Value	*p*_interaction_^a^
	HR (95% CI)		HR ^a^ (95% CI)		
Risk of pneumonia					
LABA					0.558
No	1.44 (1.37, 1.52)	<0.001	1.14 (1.07, 1.21)	<0.001	
Yes	1.76 (1.26, 2.45)	0.001	1.3 1(0.90, 1.90)	0.161	
SABA					0.358
No	1.43 (1.34, 1.52)	<0.001	1.13 (1.05, 1.21)	0.001	
Yes	1.57 (1.39, 1.76)	<0.001	1.23 (1.07, 1.40)	0.003	
LAMA					0.920
No	1.43 (1.35, 1.51)	<0.001	1.14 (1.07, 1.21)	<0.001	
Yes	1.88 (1.54, 2.30)	<0.001	1.28 (1.01, 1.61)	0.042	
ICS					0.262
No	1.42 (1.34, 1.51)	<0.001	1.12 (1.05, 1.20)	0.001	
Yes	1.56 (1.40, 1.75)	<0.001	1.24 (1.09, 1.41)	0.001	

^a^ Adjusted for propensity score (continuous), cardioversion procedure, and amiodarone use, which was calculated as a time-varying covariate.
